# Treatment of atrophic cutaneous leishmaniasis scar using autologous fibroblasts and keratinocytes (a case report and literature review)

**Published:** 2010

**Authors:** Mohammad Ali Nilforoushzadeh, Mohhamad Hossein Nasr Esfahani, Mehr Afarin Fesharaki, Amir Hossein Siadat, Nazli Ansari, Elahe Haft Baradaran

**Affiliations:** a Dermatologist, Associated Professor of Dermatology, Head of Skin Diseases and Leishmaniasis Research Center (Sedigheh Tahereh), Isfahan University of Medical Sciences, Isfahan, Iran; b Center for Research and Training in Skin Diseases and Leprosy, Tehran University of Medical Sciences, Tehran, Iran; c Embryologist, Rooyan Institute, Jahad Daneshgahi, Tehran, Iran; d Department of Physiology, Isfahan University of Medical Sciences, Isfahan, Iran; e Dermatologist, Researcher in Skin Diseases and Leishmaniasis Research Center (Sedigheh Tahereh), Isfahan University of Medical Sciences, Isfahan, Iran; f General Physician, Researcher in Skin Diseases and Leishmaniasis Research Center (Sedigheh Tahereh), Skin Disease and Stem Cell Research Center, Isfahan University of Medical Sciences, Isfahan, Iran; g Skin Disease and Leishmaniasis Research Center (Sedigheh Tahereh), Isfahan University of Medical Sciences, Isfahan, Iran

**Figure 1:**
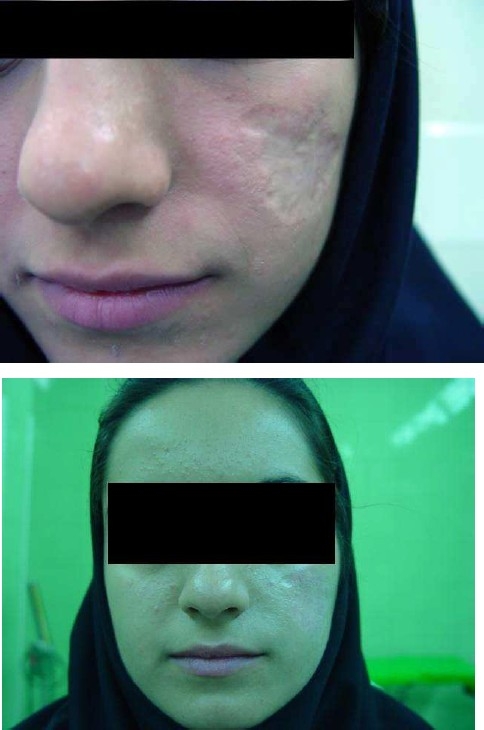
The appearance of scar before and after treatment

Cutaneous leishmaniasis is an endemic disease of Iran. Unfortunately, there is no definite treatment for this disease.^1^ We report a 21-year-old woman who was infected with cutaneous leishmaniasis about 16 years ago with disfiguring scar (Figure 1). Different methods of resurfacing were taken without significant results. To repair the scar, at first, a biopsy was performed from retroauricular area and was sent for Rooyan where culture of fibroblasts was performed. A mixture of 20 millions fibroblasts in 1 cc of serum was injected beneath the scar area with about 100% correction. After 2 months, fibroblast suspension was injected again. The scar area was then dermabraded until blood oozing was occurred. The dermabraded area was covered with a thin layer of Fibrin Glue, fibroblast and keratinocyte suspension. The dressing was removed 2 weeks later. The appearance of scar was significantly improved at 3-month follow-up. According to two blinded investigators and the patient herself, there was at least 80% and 90% improvement, respectively, in the cosmetic appearance of the scar (Figure 1).

Leishmaniasis scars are usually depressed and atrophic. Patients affected by these scars usually have psychosocial and cosmetic complains.^2^ The result at 3 months follow up was very interesting and the cosmetic appearance of leishmaniasis scar was at least 90% improved according to the patient and investigators. More prolonged studies on further cases are recommended for better evaluation of this method in the treatment of atrophic scars.
